# Dental Students' Didactic and Psychomotor Skills Performance in Dental Anatomy and Preclinical Operative Dentistry Courses in a Saudi Governmental School

**DOI:** 10.1155/2021/7713058

**Published:** 2021-12-02

**Authors:** Afnan O. Al-Zain, Adel M. Abdel-Azim, Hisham I. Othman

**Affiliations:** ^1^Operative and Esthetic Dentistry Division, Restorative Dentistry Department, Faculty of Dentistry, King Abdulaziz University, P.O. Box 80209, Jeddah 21589, Saudi Arabia; ^2^Oral Pathology Department, Faculty of Dentistry, Ain Shams University, El-Khalyfa El-Mamoun, Street Abbasya, Cairo, Egypt; ^3^Dental Anatomy and Oral Histology Division, Oral Diagnostic Science Department, Faculty of Dentistry, King Abdulaziz University, P.O. Box 80209, Jeddah 21589, Saudi Arabia

## Abstract

**Background:**

Knowledge and psychomotor skills are essential in dental education. The aims were to (1) investigate the correlation between dental students' didactic and psychomotor skills performance in the dental anatomy and preclinical operative dentistry courses and (2) explore the impact of gender on students' performance in both courses.

**Materials and Methods:**

A retrospective cohort study was performed on dental students' (164 students; 72 males and 92 females) dental anatomy and preclinical operative courses scores of the same class over 2 years (2018–2020). Didactic and practical scores were collected. The didactic scores included examinations. Practical scores included tooth wax carving for the dental anatomy course and class II cavity preparations and restorations for the preclinical operative. Student's *t*-test and ANOVA were used to analyze the difference between the didactic and psychomotor skills scores of both courses and genders. Pearson's correlation coefficient was used to explore correlations (*p* < 0.05).

**Results:**

Moderate, positive, and significant correlations were found between didactic scores in both courses and between dental anatomy's didactic and psychomotor skills. A weak, positive, and significant correlation existed between the preclinical operative didactic and psychomotor silks (*p* < 0.05). Females' didactic performance was significantly better than males. Gender had a significant, positive, and moderate correlation in the dental anatomy course, but moderate-weak in preclinical operative (*p* < 0.05).

**Conclusion:**

Students' didactic and psychomotor performance correlations in dental anatomy and preclinical operative courses were positive. The correlation was moderate and weak and varied by course. Gender had a significant impact on student performance and varied by procedure and courses investigated.

## 1. Introduction

The acquisition of various skills is an integral part of dental education [1]. Psychomotor skill is an essential skill that dental students need to develop in addition to scientific knowledge [1–3]. Before starting treating patients in clinics, students must be competent by cultivating their carving, tooth preparation, and restorative technique skills, among other procedures in the preclinical laboratory [1, 2].

Practicing dental carving of teeth is an essential aspect of undergraduate dental education because it helps develop their psychomotor skills [4, 5]. The training of wax carving in dental anatomy course by carving of natural teeth dimensions or missing portions of teeth placed in a dental articulator has played a significant part in the students' knowledge and implementation in other courses where such information is necessary [6, 7]. The morphological shape of the reconstructed teeth must be accurate to restore proper dental function [8–10]. Dental students should be able to identify the type, shape, and feature of each tooth and to maintain the esthetic and physiological character of the patient's stomatognathic system [4, 5, 7].

The preclinical operative dentistry course is of similar importance where various procedures are practiced on artificial teeth placed inside a patient simulator. In the preclinical simulation laboratory, students learn different procedures, including different cavity preparation classes and designs, restoring cavities with composite and amalgam restorative materials, among other adjunctive procedures such as base, rubber dam, and matrix band and wedge application, all of which require a high degree of psychomotor ability [11–13].

Many dental schools use standardized admission tests and the grade point average as admission tools [14–16]. Psychomotor skills have value as a tool for screening candidates for admission to some dental schools [17–20]. However, manual dexterity can be enhanced after intensive training [21]. Some students cannot advance through further clinical practices to treat patients because of their low performance in preclinical courses [22]. It is questionable if manual expertise and dental knowledge are appropriate metrics for dental school admission or predictive indicators relevant to professional success [23]. The overall assessment of each course is the cumulative exam scores of both knowledge and practical skills, though independently scored. A high didactic and low practical score, for example, would give an average degree, which could influence the student's actual achievement.

Studies were carried out to identify associations between didactic achievements and preclinical scores of dental students [1, 24, 25]. It was proposed that the scope of dental procedures needs a wide range of knowledge and skills and that manual dexterity is just a part of that advanced dental practices become entirely able to train during the dental curriculum [24]. Several studies investigated the relationship between didactic and psychomotor skills scores; others investigated the correlation of students' performance among different subjects [1, 22, 25, 26]. Studies showed a weak correlation between students' didactic course scores and their psychomotor skill scores in four different courses and that the correlation was course dependent and that the psychomotor skills performance could not be predicted from the didactic scores [1, 25]. A weak, positive, and significant correlation was found between students' soap carving scores and their clinical operative scores [26]. These studies included the overall practical scores and were not specific to certain procedures.

Few studies examined gender disparities in dental student outcomes, most of which were performed in the United States [9, 13, 21]. The few outcomes ranged from little difference to males doing well [12, 13, 21]. Another study reported that gender did not have a significant impact on state board performance [27]. A different study reported that females showed a significant correlation between didactic and clinical performance in some courses [1]. Notably, no existing researches have examined gender differences in dental student achievement in didactic versus psychomotor-dental anatomy and operative dentistry courses. Therefore, there is a gap in knowledge regarding the ability to predict students' practical performance and the effect of gender on the didactic and psychomotor skills performance in the dental anatomy and preclinical operative dentistry courses.

The study aimed to (1) investigate the correlation between dental students' didactic and psychomotor skills performance in dental anatomy and preclinical operative dentistry courses and (2) explore gender impact on the didactic and psychomotor skills performance in dental anatomy and preclinical operative dentistry courses. The null hypotheses were as follows: (1) there is no significant correlation between dental students' didactic and psychomotor skills performance in dental anatomy and preclinical operative dentistry courses and (2) gender does not significantly impact the didactic and psychomotor skills performance in dental anatomy and preclinical operative dentistry courses.

## 2. Materials and Methods

### 2.1. Study Design

This study was reviewed and approved by the Research Ethics Committee at the Faculty of Dentistry, King Abdulaziz University, proposal #177-12-20. A retrospective cohort study was performed on dental students in the same class over two years. The dental program at King Abdulaziz University Faculty of Dentistry (KAUFD) is a six-year program, and information was obtained from the second-and third-year students.

Didactic and practical scores were obtained from two courses; the dental anatomy course (2018–2019) was offered in the program's second year. The preclinical operative dentistry course (2019–2020) was provided in the third year. This study included scores of all students in the second-year class (2018–2019) that passed all subjects and moved on to the third year (2019–2020), whether males or females. Exclusion criteria included students who transferred to a different school after the second year or failed in subjects and did not move on to the third year. Both genders' students' ages ranged from 19 to 23 years. The total number of students' scores included in this study was 164: 72 males (44%) and 92 females (56%). Each course consisted of a didactic and a practical component. The dental anatomy course consists of 55% didactic and 45% laboratory practical training in wax carving of different teeth. The preclinical operative dentistry course consisted of 50% didactic and 50% laboratory practical training in the preclinical simulation phantom laboratory of various procedures, including preparing different cavity classes and designs, amalgam, composite restorations, and adjunctive procedures.

The didactic scores were obtained for each course and comprised the series of theoretical tests conducted during the academic year and included the quizzes, midyear, and final examinations. Regarding the practical procedures scores, faculty members assigned to each course were involved in calibration sessions at the beginning of each academic year. The faculty involved in the courses were male and female senior and junior faculty members. The senior faculty were specialists and consultants in their respected departments. The junior faculty were teaching assistants in their respected departments. The calibration session for each course was performed based on a rubric, which is the same rubric used by students during the academic year. The calibration included several stations of the different procedures the students perform throughout the year. When a station received inconsistencies in scoring by faculty, that station was revisited, discussed until a consensus is reached, and another calibration session was conducted to ensure all faculty are calibrated.

During the scheduled laboratory sessions, students worked on the assigned procedure with the faculty in the same lab but without direct one to one observation from the faculty. This allowed us to eliminate the stress that may be caused when the student's work is being directly observed by a faculty member. The procedure was not timed but needed to be completed in the same session. After the student completed the procedure, the student would approach the faculty for the procedure to be evaluated. The assessment during laboratory sessions was according to a criterion defined in rubrics distributed to students at the beginning of the year. The faculty assigned the practical scores after evaluating students' hand-eye and visual-spatial synchronization in a practical assessment. Multiple faculty members evaluated each procedure, and the average score was given to the student. The dental anatomy course procedures evaluated and incorporated in the study included the wax carving scores of the practical procedural experience requirements and the preclinical simulated competency examinations for different teeth. The preclinical operative dentistry course procedure scores included in this study were class II cavity preparation and class II composite restoration practical procedural experiences requirements and the preclinical simulated competency examinations. The examinations of both courses included assessments of specified learning outcomes for each course based on a blueprint.

The dental anatomy assessment form included a rubric of the different parameters assessed. The assessed parameters included the labial/buccal, lingual/palatal, proximal, incisal aspects, and root features of the carved tooth, occlusion and wax polishing, and the overall waxing skills. Each item included multiple criteria that were assessed. For example, the labial aspect of an upper canine included an evaluation of (1) accurate measurements, (2) crown shape, (3) position of the contact area, and (4) labial cusp ridge. Each item was then scored as either excellent, good, or poor. A total score was summed, representing the individual procedure score (Appendix A in Supplementary Materials is a sample of the used rubric).

The preclinical operative assessment included assessing different parameters according to cavity preparation and restoration rubrics and assessment sheets. The class II cavity preparation for composite rubric and assessment sheet included the outline form, primary retention form, primary resistance form, and finishing of walls. Each item included an evaluation of different criteria. For example, the outline form included an assessment of the cavity (1) width, (2) extension, and (3) depth. Each criterion was scored with 2 (all criteria fulfilled), 1 (one or more of the criteria fulfilled), and 0 (none of the criteria fulfilled). Then, the given scores were summed, and a final score and percentage were given (Appendix B in Supplementary Materials). Class II composite restoration assessment included different parameters: margins, contact and contour, surface texture, and occlusion checking. Each item included an evaluation of different criteria. For example, assessing the margins included (1) sealing and integrity and (2) overhang (Appendix C in Supplementary Materials). For the class II cavity preparation and composite restoration assessed, each criterion was scored with 2 (all criteria fulfilled), 1 (one or more of the criteria fulfilled), and 0 (none of the criteria fulfilled). Then, the given scores were summed, and a final score and percentage were given. For all preclinical operative procedures, critical mistakes were identified in the rubric to students.

Faculty members of each course assessed the requirements based on the designated procedure rubric. For the practical examinations, three faculty members evaluated the exams, where each faculty member evaluated the student individually, and the average score for the three faculty members was calculated for each student. For cases where the first attempt failed, the first test marks were considered rather than the makeup examination marks. The scores were converted on a continuous scale to 100 to standardize the didactic and psychomotor skills procedure scores. The scores of the same student were compared between the courses. Moreover, the didactic and practical scores of each course for each student were compared to each other. Since male and female dental students at KAUFD are taught independently during didactic lectures and laboratory sessions, the scores were analyzed together and separately to see the effect of gender on their performance.

### 2.2. Statistical Analysis

Data was collected and subjected to statistical analysis using Jamovi and R software. Descriptive statistics of the scores obtained in the dental anatomy and the preclinical operative dentistry courses were presented. The Kolmogorov-Smirnov and Shapiro-Wilk tests were used to test for normality in the distribution of the data. Student's *t*-test was used to compare the dental anatomy and preclinical operative dentistry course's didactic scores. One-way analysis of variance (ANOVA) followed by Tukey post hoc test was used to compare the psychomotor scores (psychomotor-dental anatomy, psychomotor-cavity preparation, and psychomotor-composite restoration). Student's *t*-test was used to test the difference between the didactic and psychomotor skills scores of both courses between male and female students. Pearson's correlation coefficient was used to test correlations between the didactic and psychomotor skills for each course. It was also used to test psychomotor skills correlations between males and females combined and separately for each course. All statistical analyses were made under the level of alpha = 0.05.

## 3. Results

### 3.1. Didactic and Psychomotor Skill Performance

#### 3.1.1. Didactic and Psychomotor Scores

For male and female scores combined, the mean didactic-dental anatomy scores were 72.4% (8.5), and the mean didactic-preclinical operative dentistry scores were 79.5% (7.7) and found no significant differences. The mean psychomotor-dental anatomy scores were 76.8% (10.0), the mean psychomotor-cavity preparation scores were 82.6% (5.9), and the mean psychomotor-composite restoration scores were 81.3% (5.5). Significant differences between psychomotor-dental anatomy and psychomotor-cavity preparation were found between psychomotor-dental anatomy and psychomotor-composite restoration scores (*p* < 0.001). Regarding the dental anatomy course, no significant differences were found between the didactic and psychomotor performance. Concerning the preclinical operative course, significant differences were found between the didactic and psychomotor-composite restoration performance scores.

#### 3.1.2. Effect of Gender on the Didactic and Psychomotor Scores

**Figure 1:**
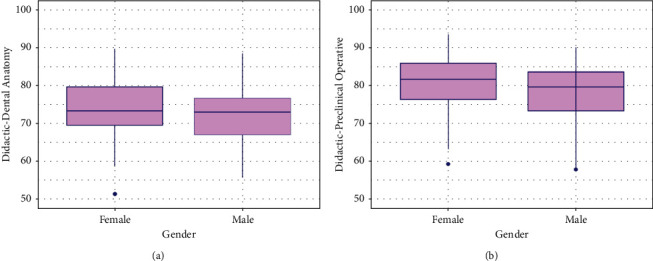
Box plots of the dental anatomy and preclinical operative dentistry courses didactic scores for male and female students. (a) Dental anatomy didactic scores for male and female students. (b) Preclinical operative dentistry didactic scores for male and female students.

**Figure 2:**
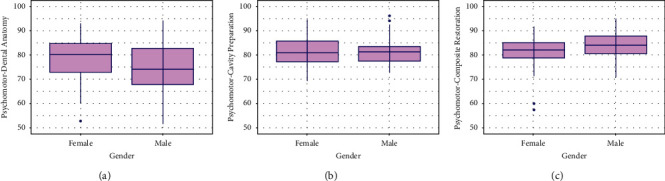
Box plots of the dental anatomy and operative psychomotor silks for male and female students. (a) Dental anatomy psychomotor skills for male and female students. (b) Cavity preparation psychomotor skills for male and female students. (c) Composite restoration psychomotor skills for male and female students.

It was evident that females had significantly higher score percentages in didactic-dental anatomy (*p*=0.024) and didactic-preclinical operative dentistry courses (*p*=0.015) than males (Figure 1). The score percentages of each course's psychomotor skills were different between male and female students (Figure 2). Female psychomotor-dental anatomy scores were significantly higher than males (*p*=0.029) (Figure 2(a)). There was no significant difference in psychomotor-cavity preparation scores between genders (*p*=0.767) (Figure 2(b)). However, male psychomotor-composite restoration scores were significantly higher than females' (*p*=0.001) (Figure 2(c)).

### 3.2. Correlations

#### 3.2.1. Correlations of the Didactic and Psychomotor Skills Scores

Correlations between the didactic and psychomotor skills scores of the dental anatomy and preclinical operative dentistry courses for males and females combined showed interesting findings (Table 1). The didactic score correlations between courses and psychomotor skills showed that the correlation between didactic-dental anatomy and psychomotor-dental anatomy scores was moderate, positive, and significant. The correlation between didactic-dental anatomy and didactic-preclinical operative dentistry was moderate, positive, and significant. The correlation between didactic-preclinical operative dentistry and psychomotor-cavity preparation and didactic-preclinical operative dentistry with psychomotor-composite restoration scores was weak, positive, and significant.

Regarding the psychomotor skill scores correlations between courses, it was found that the correlation between psychomotor-dental anatomy scores and psychomotor-cavity preparation was moderate, positive, and significant. The correlation between psychomotor-dental anatomy and psychomotor-composite restoration scores was weak, positive, and significant. The correlation between psychomotor-cavity preparation and psychomotor-composite restoration scores was weak, positive, and significant.

#### 3.2.2. Correlations of the Didactic and Psychomotor Scores between Genders

Correlations among the didactic and psychomotor skills scores for the dental anatomy and preclinical operative dentistry courses per gender showed interesting findings (Table 2). For male and female students, the correlation between didactic-dental anatomy with psychomotor-dental anatomy scores and didactic-dental anatomy with didactic-operative dentistry scores was moderate, positive, and significant. On the other hand, the didactic-preclinical operative correlation with psychomotor-cavity preparation scores was weak, positive, and significant for male and female students. The correlation of didactic-preclinical operative with psychomotor-composite restoration scores was moderate, positive, and significant for males and weak, positive, and significant for females.

The psychomotor skills scores correlate with psychomotor-dental anatomy and psychomotor-cavity preparation, and the correlation between psychomotor-dental anatomy and psychomotor-composite restoration scores were moderate, positive, and significant for male and female students. However, the correlation between psychomotor-cavity preparation and psychomotor-composite restoration was weak, positive, and significant for males and was moderate, positive, and significant for females.

## 4. Discussion

This study aimed to explore whether there is a relationship between dental students' didactic and psychomotor skills achievements in dental anatomy and preclinical operative dentistry courses and to investigate if gender influences the associations mentioned above. Both courses have didactic theoretical components in the form of lectures, which are assessed in different methods, including quizzes, midyear, and final examinations. In the second year of the dental program at KAUFD, the practical session component of the dental anatomy course, students learned how to handle different dental instruments and wax carving different teeth in their natural dimensions. In the third year, students were taught how to perform various procedures in the preclinical operative dentistry course on artificial plastic teeth placed in a phantom head patient simulator. The procedures included cavity preparations of different classes and designs, restoring various cavity preparations with different restorative materials such as amalgam and resin-based composites, and adjunctive procedures including matrix band and wedge application, rubber dam application, and base application. Since male and female dental students at KAUFD are taught independently during didactic lectures and laboratory sessions, the scores were analyzed together and separately to see the effect of gender on their performance.

In our study, scores of specific preclinical operative procedures were included to standardize the skill comparisons between both subjects. The class II composite resin restoration procedure was included in the study because it involves skills similar to the wax carving procedure. Class II cavity preparation procedure scores were included because they demonstrate the development of a different set of skills, including stability and control during cutting.

The combined male and female didactic score percentages showed that there were no significant differences between the courses. In addition, a moderate, positive significant correlation between didactic-dental anatomy course and the investigated didactic-preclinical operative dentistry course scores in the following year has been achieved. This can indicate that the students' didactic achievement is not impacted by the type of courses investigated.

On the other hand, significant differences were observed between psychomotor-cavity preparation and psychomotor-composite restoration and between didactic-preclinical operative and psychomotor-composite restoration scores. This can indicate that the students' psychomotor achievement is influenced by the type of procedure and course investigated. The correlations of the combined male and female didactic-dental anatomy and psychomotor-dental anatomy skills scores were moderate, positive, and significant. However, the correlations of the combined male and female didactic-preclinical operative and psychomotor-cavity preparation and psychomotor-composite restoration skills scores were weak, positive, and significant. Therefore, the first null hypothesis was rejected. This positive correlation indicates that the proper academic performance of students was achieved with good practical dental skills. Theoretical knowledge can assist students in practical sessions to achieve better results. Students who do not know or recognize the teeth's morphological details would probably have difficulty carving or restoring the correct morphological details in wax or even building the right anatomical and physiological elements of various restorations. Guidelines for dental carving had a significant role in coaching students by improving their manual dexterity for other branches of science that need such abilities. It would not be possible for students who do not identify the various cavity preparation stages or prepare different cavity designs for various dental restorations to operate on patients or perform their treatment correctly and according to the appropriate requirements.

In our study, the students' wax carving scores could significantly predict their practical scores in preclinical operative dentistry course, especially in cavity preparation skills showing a positive correlation. This previous result clarified that psychomotor skills play a vital role and can be trained during the dental anatomy course. On the other hand, the dental anatomy's psychomotor skills show a weak positive correlation with psychomotor-composite restoration skills. This could be explained by the distinct nature of the used carving materials' properties for building amalgam and composite restoration versus wax carving. Also, preclinical operative procedures are performed in a simulated phantom head and not on a benchtop as in the dental anatomy course, impacting the students' performance. Our results partially agreed with other studies where a weak positive correlation was found [1, 25, 26]. In addition, our results agreed to a study that showed that the correlation between didactic and psychomotor performance is course-dependent and that a correlation existed in the dental anatomy course [25].

Results showed that gender had an impact on performance in didactic scores and specific psychomotor procedures. Females significantly outperformed their male colleagues in the didactic component of both courses and the psychomotor-dental anatomy component. However, males significantly outperformed females in psychomotor-composite restoration per the course scores. Nevertheless, gender did not significantly impact psychomotor-cavity preparation skills scores. Therefore, the second null hypothesis was rejected. This may be explained by the different skill sets developed between genders. The possible role of gender is becoming increasingly more significant when evaluating the academic performance of students. Studies have found that, in exams, female students of medical colleges achieved better than male students [4, 6, 8]. The matter of gender disparities in dental exam performance is still unclear. However, other studies have proved that most males are more competitive than females [12, 13, 21]. Other researchers have established no gender disparity in dental students' achievement. A general policy of gender classification is a unique characteristic of higher education in Saudi Arabia, and admission to health sciences faculties relies primarily on the scores in addition to the desires of students. Female students performed better on this exam than males, getting higher scores, and consequently, a strong competition between females may have been created. This previous explanation supports our results where the female students have better performance counterparts in didactic-dental anatomy and operative dentistry courses. Our study agreed to some extent with a study that showed no significant differences between genders after admission to the dental school in terms of technical skills development [17]. One of the limitations of the study is that faculty members knew the students and future work would include masking the names of the students to eliminate bias.

It is noteworthy to mention that academic success is dynamic and complex and that environmental and psychological factors may contribute to the student's performance in the different courses. Therefore, future research should concentrate on aspects such as the quality of the educational process, the nature and duration of psychomotor learning tasks, and environmental and psychological factors that could impact the learning process and compare these aspects among dental schools. In addition, confounding factors, such as the artistic nature and skills of each student, faculty supervision, and guidance, need to be considered and investigated so different aspects are studied, and the differences in the performance of dental students in various dental courses can be better understood. The study results could constitute a preliminary analysis of a multicenter study for governmental and private dental schools across the country to be conducted in the future. In addition, it would be valuable to carry out this same investigation with other groups of students from different generations to observe if the differences in gender performance found in this group are also observed in another group to draw conclusions about which gender performed better.

## 5. Conclusions

The correlation between dental students' didactic and psychomotor skills in dental anatomy and preclinical operative dentistry courses was significant, with a moderate to weak positive correlation that varied by course and psychomotor procedure in a governmental dental school. Gender had a significant impact on the didactic performance and varied in terms of the psychomotor skills performance of the dental anatomy and preclinical operative dentistry courses.

## Figures and Tables

**Table 1:** Pearson's correlation matrix and *p* values for dental anatomy and preclinical operative dentistry didactic and psychomotor skills percentage scores for male and female students combined.

	Didactic-dental anatomy	Didactic-preclinical operative	Psychomotor-dental anatomy	Psychomotor-cavity preparation	Psychomotor-composite restoration
Didactic-dental anatomy	—				

Didactic-preclinical operative	*R* = 0.606 (*p* < 0.001)	—	—		

Psychomotor-dental anatomy	*R* = 0.539 (*p* < 0.001)		—		

Psychomotor-cavity preparation	—	*R* = 0.169 (*p*=0.031)	*R* = 0.434 (*p* < 0.001)	—	

Psychomotor-composite restoration	—	*R* = 0.200 (*p*=0.010)	*R* = 0.253 (*p*=0.001)	*R* = 0.264 (*p* < 0.001)	—

**Table 2:** Pearson's correlation matrix and *p* values of dental anatomy and preclinical operative dentistry didactic percentage scores with psychomotor skills percentage scores for male (M) and female (F) dental students.

	Didactic-dental anatomy	Didactic-preclinical operative	Psychomotor-dental anatomy	Psychomotor-cavity preparation	Psychomotor-composite restoration
Didactic-dental anatomy	—				
	—				
Didactic-preclinical operative	M: *R* = 0.545 (*p* < 0.001)	—	—		
	F: *R* = 0.632 (*p* < 0.001)	—	—		
Psychomotor-dental anatomy	M: *R* = 0.585 (*p* < 0.001)	—	—		
	F: *R* = 0.469 (*p* < 0.001)	—	—		
Psychomotor-cavity preparation	—	M: *R* = 0.176 (*p* < 0.001)	M: *R* = 0.558 (*p* < 0.001)	—	
	—	F: *R* = 0.163 (*p* < 0.001)	F: *R* = 0.360 (*p* < 0.001)	—	
Psychomotor-composite restoration	—	M: *R* = 0.323 (*p*=0.002)	M: *R* = 0.337 (*p*=0.004)	M: *R* = 0.240 (*p*=0.043)	—
	—	F: *R* = 0.208 (*p* < 0.001)	F: *R* = 0.285 (*p*=0.006)	F: *R* = 0.305 (*p*=0.003)	—

## Data Availability

The grades data used to support the findings of this study are restricted by the Research Ethics Committee at King Abdulaziz University Faculty of Dentistry in order to protect student's privacy. Data are available from Dr. Afnan O. Al-Zain, alzain@kau.edu.sa, for researchers who meet the criteria for access to confidential data.

## Supplementary Materials

Supplementary Materials Appendix A: an example of the practical evaluation form of a maxillary central incisor wax carving and scoring rubric in the dental anatomy and occlusion course. Appendix B: cavity preparation for resin composite rubric and practical assessment form of class II cavity preparation in the preclinical operative and esthetic dentistry course. Appendix C: restoration rubric and practical assessment form of class II resin composite restoration in the preclinical operative and esthetic dentistry course.
