# Supplementary material for: Dental Students' Didactic and Psychomotor Skills Performance in Dental Anatomy and Preclinical Operative Dentistry Courses in a Saudi Governmental School
Source: Int J Dent. 2021 Dec 2;2021:7713058. doi: 10.1155/2021/7713058 (PMC8660179; doi:10.1155/2021/7713058)
Supplement: Supplementary Materials — Appendix A: an example of the practical evaluation form of a maxillary central incisor wax carving and scoring rubric in the dental anatomy and occlusion course. Appendix B: cavity preparation for resin composite rubric and practical assessment form of class II cavity preparation in the preclinical operative and esthetic dentistry course. Appendix C: restoration rubric and practical assessment form of class II resin composite restoration in the preclinical operative and esthetic dentistry course. [file 7713058.f1.zip › Appendix 1 (1).pdf]

Dental Anatomy and Occlusion Course

Rubric

Practical Evaluation Form / Maxillary Central Incisor Wax Carving

(2nd Year, 2020-21)

Student's Name:--

Comp No:----------------

	Student's Self evaluation			
	Criteria	Poor / 0	Good / 1	Excellent / 2
Labial Aspect	1. Accurate measurements			
	2. Crown shape (Trapezoidal)			
	3. Position of contact areas			
	4. Sharpe mesio-incisal angle, rounded disto-incisal angle			
Lingual Aspect	1. Lingual convergence			
	2. Clear marginal ridges and incisal ridges			
	3. Wide & Shallow fossa			
	4. Rounded cingulum			
Proximal Aspect	1. Crown shape (Triangular)			
	2. Labial and lingual outlines			
	3. Height of contour in cervical third			
	4.Curvature of cervical line			
Incisal Aspect	Outline features			
Root features	Overall conical shape with rounded apex			
Waxing skills	Wax finishing and polishing			
	Out of 30 / ---------------			

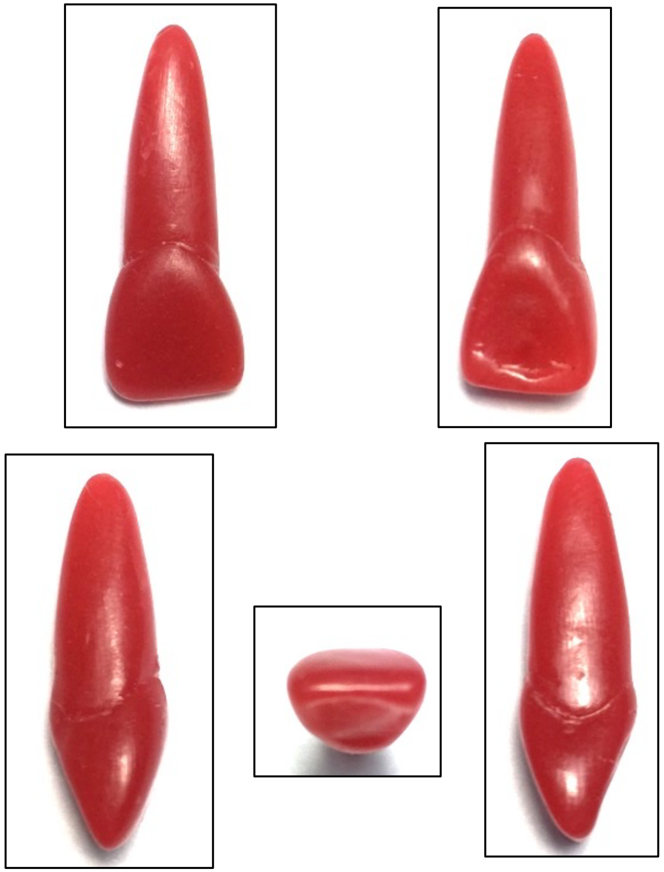

Maxillary Central Incisor Wax carving
Scoring Rubric
2nd year / 2017-18

Contents	Poor / 0	Good / 1	Excellent / 2
1. Labial Aspect	Severe deviation from criteria	Most crown and root outlines are represented	All outlines are represented properly
2. Lingual aspect	Severe deviation from criteria	Most crown and root outlines are represented	All outlines are properly represented
3. Proximal aspect	Severe deviation from criteria	Most crown and root outlines are represented	All outlines are properly represented
4. Incisal aspect	Severe deviation from criteria	Most outlines are represented	All outlines are properly represented
5. Root Features	Severe deviation from criteria	Most outlines are represented	All outlines are properly represented
E. Waxing skills	No Polishing	Moderate polished	Highly polished
