# Supplementary material for: Dental Students' Didactic and Psychomotor Skills Performance in Dental Anatomy and Preclinical Operative Dentistry Courses in a Saudi Governmental School
Source: Int J Dent. 2021 Dec 2;2021:7713058. doi: 10.1155/2021/7713058 (PMC8660179; doi:10.1155/2021/7713058)
Supplement: Supplementary Materials — Appendix A: an example of the practical evaluation form of a maxillary central incisor wax carving and scoring rubric in the dental anatomy and occlusion course. Appendix B: cavity preparation for resin composite rubric and practical assessment form of class II cavity preparation in the preclinical operative and esthetic dentistry course. Appendix C: restoration rubric and practical assessment form of class II resin composite restoration in the preclinical operative and esthetic dentistry course. [file 7713058.f1.zip › Appendix 2 (1).pdf]

## 1. Cavity Preparation for Resin Composite Rubric

| Stage | Principle | Grade | | | Total | |
|---|---|---|---|---|---|---|
| | | 2 | 1 | 0 | | |
| **Outline Form** | **Width** | **Minimal**<br>Buccolingual width = 1/4 the inter cuspal distance | **Slightly wide**<br>Buccolingual width >1/4 < 1/2 the inter cuspal distance | **Wide**<br>Buccolingual width > 2/3 the inter cuspal distance | 10% | **30%** |
| | **Extension** | **Proper**<br>Primary pits and fissure are included | **Under-extended**<br>Does not include all the primary pits and fissure | **Over-extended**<br>Extends to the cusps and marginal ridge | 10% | |
| | **Depth** | **Proper and even**<br>> 1 mm< 3 mm | **Uneven**<br>Cavity depth uneven | **Shallow/Deep**<br>< 1 mm **OR**<br>> 3 mm | 10% | |
| **Primary Resistance form** | **Pulpal and gingival floor** | **Uniform**<br>Horizontal, flat **AND** uniform | **Half is uniform**<br>Half is horizontal, flat **and/or** slanted | **Not uniform**<br>Not flat **AND** not uniform | 10% | **30%** |
| | **Wall direction** | **Converged**<br>Buccal and lingual walls converge | **Half converged**<br>Half of the buccal or lingual walls converged | **Diverged**<br>Buccal and lingual walls diverged | 10% | |
| | **Line angles** | **Slightly rounded** | **Half is rounded** | **Sharp** | 10% | |
| **Primary Retention form** | **Primary retentive features** | - Efficient undercut<br>**AND**<br>- Efficient bonding technique | - Inefficient undercut<br>**OR**<br>- Inefficient bonding technique | - Inefficient undercut<br>**AND**<br>- Inefficient bonding technique | 10% | **20%** |
| | **Each part is retentive** | **All retentive** | **Partially retentive** | **Not retentive** | 10% | |
| **Finishing of walls** | **Cavo-surface margin** | - 90 degrees for Cl I and II<br>**OR**<br>- Proper bevel when indicated in Cl III, IV, V | - Some are 90 degrees<br>**AND/OR**<br>- Narrow or ununiform bevel when indicated | - All are not 90 degrees<br>**AND**<br>- Inappropriate or NO bevel when indicated | 10% | **20%** |
| | **Walls** | **Smooth** | **Half are smooth** | **Rough** | 10% | |
| **Total** | | | | | | **100%** |

**Critical mistakes:**

1. Improper cavity depth:
   a. Less than 1 mm (repeated more than twice).
   b. More than 3 mm pulpally or axially.
2. Any damage to the adjacent tooth/teeth.

## Cavity Preparation Assessment Form

**Tooth No.: Molar (      )** **Class: II**

**Type of restoration:** ☐ **Composite** ☐ **Amalgam**

| | | | |
|---|---|---|---|
| **Ethical conduct and professionalism** | ☐ **Yes** | ☐ **No** | Student shows respect towards staff, and colleagues. Student follows faculty directives, student wears appropriate professional attire, presents only his/her work (no cheating). |
| **Communication skills** | ☐ **Yes** | ☐ **No** | Student communicates efficiently with instructors using clear words and logical sequence. |
| **Cubic/tray organization** | ☐ **Yes** | ☐ **No** | Student organizes the instruments in the proper sequence in his/her tray. Surfaces are clean and properly wrapped. No visible littering. |

*0 none of criteria fulfilled, 1 min one criteria fulfilled, 2 some of criteria fulfilled, 3 all criteria fulfilled*

| **Steps and procedures** | **Weight %** | **Student self-evaluation** | | | **Instructor evaluation** | | | **Feedback** | | |
|---|---|---|---|---|---|---|---|---|---|---|
| | | 0 | 1 | 2 | 0 | 1 | 2 | | | |
| **Outline Form** | **30** | | | | | | | W/ | E/ | D/ |
| **Resistance Form** | **30** | | | | | | | F/ | W/ | A/ |
| **Retention Form** | **20** | | | | | | | R/ | E/ | |
| **Finishing of walls** | **20** | | | | | | | C/ | W/ | |

| **Date** | **Faculty Stamp and Signature** | **Score** |
|---|---|---|
| | | **/10** |

☐ Level C - Basic Ability

☐ Level B - Competent Ability

☐ Level A - Proficient Ability
