# Supplementary material for: Dental Students' Didactic and Psychomotor Skills Performance in Dental Anatomy and Preclinical Operative Dentistry Courses in a Saudi Governmental School
Source: Int J Dent. 2021 Dec 2;2021:7713058. doi: 10.1155/2021/7713058 (PMC8660179; doi:10.1155/2021/7713058)
Supplement: Supplementary Materials — Appendix A: an example of the practical evaluation form of a maxillary central incisor wax carving and scoring rubric in the dental anatomy and occlusion course. Appendix B: cavity preparation for resin composite rubric and practical assessment form of class II cavity preparation in the preclinical operative and esthetic dentistry course. Appendix C: restoration rubric and practical assessment form of class II resin composite restoration in the preclinical operative and esthetic dentistry course. [file 7713058.f1.zip › Appendix 3 (1).pdf]

1. Amalgam, Resin Composite and Temporary Restorations Rubric

Parameter	Description	Grade			Total	
		2	1	0		
**Margins**	**Sealing and Integrity**	All margins sealed with no ditch	-	Submargins all around the restoration	10%	**20%**
	**Overhang**	No	-	Yes	10%	
**Contour and contact**	**Proximal**	Properly contoured	Partially contoured	Flat	10%	**30%**
	**Occlusal**	Properly contoured	Partially contoured	Flat	10%	
	**Contact***	Tight	Light	Open	10%	
**Anatomy**	**Cusps**	All built	Some built	Flat cusps	10%	**20%**
	**Pits and fissures**	- Created<br>- Original depth	- Partially created<br>- Shallow (under carving)	- Flat<br>- Deepened (over carving)	10%	
**Surface texture**	**Finishing (Smooth and no flashes)**	All smooth	Partially smooth	Rough	10%	**20%**
	**Polishing****	Lustrous	Partially lustrous	Matt	10%	
**Checking of Occlusion**	**Relation to the Opposing**	Proper occlusion	-	Premature contact (presence of high spots)	10%	**10%**
**Total**						**100%**

***Contact is examined by dental floss.**
****For Resin Composite restorations.**

Critical mistakes:

1. Improper condensation for amalgam.
2. Improper packing of resin composite.

Restoration Assessment Form

Tooth No.: Molar (   ) **Class: II**

Type of restoration: ☐ **Composite** ☐ **Amalgam**

**Ethical conduct and professionalism**	☐ **Yes**	☐ **No**	Student shows respect towards staff, and colleagues. Student follows faculty directives, student wears appropriate professional attire, presents only his/her work (no cheating).
**Communication skills**	☐ **Yes**	☐ **No**	Student communicates efficiently with instructors using clear words and logical sequence.
**Cubic/tray organization**	☐ **Yes**	☐ **No**	Student organizes the instruments in the proper sequence in his/her tray. Surfaces are clean and properly wrapped. No visible littering.

0 none of criteria fulfilled, 1 min one criteria fulfilled, 2 some of criteria fulfilled, 3 all criteria fulfilled

**Steps and procedures**	**Weight %**	**Student self-evaluation**			**Instructor evaluation**			**Feedback**		
		0	1	2	0	1	2			
**Margins**	**20**							S/	O/	
**Contour/Contact**	**30**							P/	O/	C/
**Anatomy**	**20**							C/	P/	
**Surface texture**	**20**							F/	P/	
**Occlusion**	**10**							O/		

**Date**	**Faculty Stamp and Signature**	**Score**
		**/10**

☐ Level C - Basic Ability

☐ Level B - Competent Ability

☐ Level A - Proficient Ability
